# Young people’s views about the use of celebrities and social media influencers in gambling marketing

**DOI:** 10.1093/heapro/daae012

**Published:** 2024-02-11

**Authors:** Hannah Pitt, Simone McCarthy, Melanie Randle, Mike Daube, Samantha L Thomas

**Affiliations:** Institute for Health Transformation, Deakin University, Locked bag 20000, Geelong, Vic 3220, Australia; Institute for Health Transformation, Deakin University, Locked bag 20000, Geelong, Vic 3220, Australia; Faculty of Business and Law, University of Wollongong, Wollongong, Australia; Faculty of Health Sciences, Curtin University, Perth, Australia; Institute for Health Transformation, Deakin University, Locked bag 20000, Geelong, Vic 3220, Australia; Curtin School of Population Health, Curtin University, Perth, Australia

**Keywords:** gambling, marketing, children, young people, advertising, social media, celebrity, social media influencers

## Abstract

Young people’s exposure to gambling marketing has had a clear impact on their gambling attitudes, risk perceptions and consumption intentions. Celebrities and social media influencers (SMIs) are increasingly used by the gambling industry in a wide range of promotions. While there is evidence that these types of promotions are influential in shaping young people’s attitudes towards other harmful products, there is limited evidence in relation to gambling. Qualitative focus groups (*n* = 22) with *n* = 64, 12–17 year olds were conducted in Australia. These investigated young people’s exposure to celebrity and SMI marketing for gambling and the influence they perceived this marketing had on young people’s gambling attitudes. Reflexive thematic analysis was used to construct four themes from the data. First, young people perceived that celebrities and SMIs created additional appeal and recall of gambling advertisements because they were attention grabbing and familiar. Second, young people thought that celebrities and SMIs increased the trust, legitimacy and social acceptance of gambling. Third, the use of celebrities and SMIs lowered the perceptions of risk associated with gambling. Lastly, there were suggestions to reduce the impact of celebrity and SMI gambling promotions on young people, such as bans and restrictions. This study highlights the importance of a comprehensive approach to preventing young people’s exposure to gambling marketing, that not only considers imposing stronger regulations to restrict the way the gambling industry is allowed to promote its products, but also aims to counter the novel ways the gambling industry attempts to appeal to children and young people.

**Contribution to Health Promotion**

- Provides insights regarding how young people interact with gambling marketing that features celebrities and social media influencers (SMIs).
- Demonstrates that young people perceive that these forms of marketing increase the trust, credibility and legitimization of gambling.
- Shows that young people understand the commercial and harmful nature of such endorsements, and are critical of celebrities and SMIs who engage in promotions for a range of gambling products.

## BACKGROUND

The commercial gambling industry is one of the most innovative health-harming industries of modern times, posing a significant threat to public health (Badu *et al*., 2023; Glozah *et al*., 2023; Thomas *et al*., 2023a). While the commercial gambling industry operates from a similar playbook to other health-harming industries such as tobacco and alcohol (Cowlishaw and Thomas, 2018; de Lacy-Vawdon *et al*., 2023), governments have rarely recognized the tactics of the gambling industry as a public health issue (van Schalkwyk *et al*., 2021; Thomas *et al*., 2023a). Consequently, there have been limited efforts outside of personal responsibility strategies to protect communities from harm (Miller *et al*., 2016; van Schalkwyk *et al*., 2021; Arnot *et al*., 2023d; Badu *et al*., 2023). The absence of strong global regulatory frameworks and conventions has also enabled the gambling industry to develop a range of novel products with widespread appeal to a new generation of younger gamblers, particularly when aligned with popular activities such as sport (Sichali *et al*., 2023; Thomas *et al*., 2023e). Digital transformations have revolutionized how the gambling industry is able to ‘*promote its products, gain publicity, attract new customers, shape social and cultural attitudes, and build corporate and product image and support’* (Thomas *et al*., 2023e, p. 3). These sophisticated and persuasive promotional strategies are used to create brand awareness, normalize and legitimize gambling as an everyday activity and engage new customers with an increasing array of instantaneously accessible products (Thomas *et al*., 2018; McGee, 2020; Guillou-Landreat *et al*., 2021).

Marketing strategies have also contributed to the rapid normalization of gambling for children and young people (Djohari *et al*., 2019; Nyemcsok *et al*., 2021; Riley *et al*., 2021). In most countries, children are not permitted to engage in commercial gambling until they are 18 years or older (with some exceptions for lotteries), yet they are increasingly exposed to gambling promotions in multiple media and community environments (Smith *et al*., 2020; Thomas *et al*., 2023b). This exposure has had clear impacts, including increased awareness and recall of gambling company brands (Pitt *et al*., 2017a; Nyemcsok *et al*., 2018); positive perceptions and approval of gambling as a normal or valued activity (particularly aligned with sport) (Pitt *et al*., 2016b; Djohari *et al*., 2019); reduced perceptions of the risks associated with gambling (Pitt *et al*., 2017a; Nyemcsok *et al*., 2018); and an intention to try gambling in the future (Pitt *et al*., 2017b; Nyemcsok *et al*., 2018). Most studies investigating gambling marketing have largely focused on television advertising strategies for online gambling products, particularly those aligned with major sporting codes (Pitt *et al*., 2016a; Clemens *et al*., 2017; Pitt *et al*., 2018; Labrador *et al*., 2021). However, more recent research has shown that the gambling industry uses a range of unique appeal strategies, including on social media platforms, to reach younger audiences (Pitt *et al*., 2023; Thomas *et al*., 2023b).

In response to concerns about the impact of marketing on children, the gambling industry (and those with vested interests such as broadcasters and sporting codes) claim that they are responsible corporate citizens and not directly targeting children (Sportsbet Pty Ltd, 2016; Australian Association of National Advertisers, 2018; Irish Bookmakers Association, 2021); are voluntarily enacting measures to minimize children’s exposure to incidental gambling marketing, such as the removal of marketing messages from sporting shirts (Thomas *et al*., 2023c); and only advertising on social media platforms such as TikTok with appropriate age gating technology (Chwasta, 2022; The Commonwealth, 2023). In some countries, the gambling industry uses children in their public relations strategies, including corporate social responsibility (CSR) initiatives. This includes employing agnogenic practices which are used to create ignorance or doubt, and which legitimize industry-favourable framings of gambling (van Schalkwyk *et al*., 2024).

While governments have started to acknowledge the risks associated with exposure to gambling marketing for children and young people, few have implemented comprehensive policies to protect them from this (Underwood, 2021; Walker, 2023). Parliamentary inquiries in Australia (Standing Committee on Social Policy and Legal Affairs, 2023) and the United Kingdom (All Party Parliamentary Group, 2020) have recommended complete bans or significant restrictions on gambling marketing. However, most governments have resisted enacting provisions to protect children from being exposed to the broad range of marketing strategies used by the gambling industry. Researchers have in part attributed this lack of action to powerful vested interests that financially profit from gambling and are able to influence political decisions (Thomas *et al.*, 2023e). Policy responses have mostly focused on limiting gambling marketing in certain commercial television advertising time slots, such as family–friendly hours or sports match programming (Australian Communication and Media Authority, 2020; ASA and CAP, 2022), with evaluations showing that these approaches have simply shifted gambling marketing to different time slots and platforms (Australian Communications and Media Authority, 2019).

There have also been very few efforts to regulate newer forms of gambling marketing on social media which may be particularly appealing to young people (Hörnle *et al*., 2019). Such strategies have been effective for harmful industries such as tobacco, alcohol and ultra-processed food in circumventing regulations and directly reaching young people (Freeman, 2012; Room and O’Brien, 2021; Vassallo *et al*., 2022). They include the use of celebrities and social media influencers (SMIs)—defined by Engel *et al.*, (2024). (Engel *et al.*, 2024) as *‘individuals who amass large followings on social media and exert significant influence over their audience through engaging content’* (p. 1)—in gambling marketing strategies. The use of celebrities is an effective form of marketing across a range of different products (Knoll and Matthes, 2017). This is because the symbolic meanings associated with celebrities can be transferred to the products and brands they endorse (McCracken, 1989, p. 310). Celebrity marketing of harmful products has included the use of SMIs on platforms popular with young people such as TikTok, YouTube and Instagram (Chwasta, 2022; Lee *et al*., 2023; Vassey *et al*., 2023). These types of marketing are persuasive for young people, and may influence the normalization and consumption of these products (Hendriks *et al*., 2020; Packer *et al*., 2022; Corcoran *et al*., 2023; Smith and Hilton, 2023), as well as promoting new products, and reaching young people who otherwise would not be exposed to these promotions (Campaign for Tobacco-Free Kids, 2023).

Research has shown that celebrities feature in almost half of all gambling industry social media posts (Pitt *et al*., 2023). Some regulatory bodies have proposed restrictions on celebrities in gambling promotions, stating that they may have ‘strong appeal’ to children (regardless of their appeal to adults) (ASA and CAP, 2022). Such restrictions have acknowledged that certain types of celebrities such as athletes, children’s presenters and individuals with a high following of young people on social media are ‘high risk’ in terms of their appeal to children (Committee of Advertising Practice, 2022). However, there is relatively limited research focusing specifically on the perspectives of young people in relation to celebrity and SMI promotions for gambling. This includes the types of celebrity endorsements they see in their everyday media spaces; the perceived influence different types of celebrity and SMI promotions have on young people; and the types of strategies perceived as most appropriate in responding to such promotions. The present study sought to address this gap in the literature and was guided by three research questions:

1. What are children’s and young people’s attitudes towards the use of celebrities and SMIs in gambling promotions?
2. What influence do children and young people perceive the use of celebrities and SMIs has in shaping young people’s gambling attitudes and consumption intentions?
3. What types of strategies do children and young people perceive would be useful in responding to the use of celebrities and SMIs in gambling promotions?

## METHODS

### Approach

The research in this article was part of a larger study investigating young people’s receptivity and attitudes towards gambling advertising, promotion and sponsorship. The researchers took a comprehensive public health position on gambling, which recognizes that harm from the gambling industry is caused by a range of individual, social, commercial and political determinants, and advocates that gambling policies have a key aim of protecting individuals, their communities and families from being harmed by the gambling industry, its products and practices (Thomas *et al.*, 2023e). We also took the position that young people have the right to have a say about policy decisions that impact on their health and well-being (Soraghan *et al*., 2023). As with other pressing public health issues—such as the harms from the fossil fuel industry—young people will bear many of the consequences of the decisions that are made by governments about how best to protect and promote their current and future health and well-being (Arnot *et al*., 2023a). In this context, engaging young people in discussions about gambling industry tactics is essential in developing robust responses aimed at preventing harm from the gambling industry.

### Sampling and recruitment

To be eligible for the broader study, participants were required to be 12–17 years old, reside in either Victoria or New South Wales (NSW) in Australia, have parental consent to participate, and be fluent in English. The age criterion was chosen because this is when young people become aware of marketing brands and begin to understand the persuasive intent of marketing (Hudson and Elliott, 2013). Convenience, purposive and snowball sampling techniques were used to recruit participants. Due to the age of participants, parents and guardians were the target of recruitment strategies. Recruitment notices were distributed via a range of mechanisms including using social media posts, sporting and community groups who shared information about the study with their members and parents who passed on information about the study to their social networks. Parents who were interested in their children participating contacted the research team and were provided with a plain language statement and consent form, and written parental consent was obtained, with an option for the young person to also sign the consent form. During this process, parents and their children had the opportunity to ask questions regarding their participation and the project requirements. Participants were also informed that they could withdraw at any time. Verbal consent was confirmed from all participants before the focus group discussion commenced. Before the interview began, SM outlined details about the study and the young people were reminded not to use any specific names or details about other people when discussing experiences to ensure that we respected people’s privacy. Young people were told that discussing gambling harm can be a sensitive topic and to be respectful of the feelings and opinions of other participants, especially if opinions or experiences were different from their own. They were told there were no right or wrong answers, and that they could withdraw at any time without any consequences, or turn off their camera if they did not feel comfortable having it on. They were also told they could private message the facilitator at any time during the focus group to ask questions, or to raise any concerns. All young people received a $50 voucher as a token of appreciation for taking part in the focus group. Ethical approval was provided by the Deakin University Human Research Ethics Committee [2021-304].

### Data collection

Online focus groups included 2–4 participants and were conducted between November 2021 and September 2022. Focus group methodology was used to stimulate interaction and knowledge sharing among young people, and allowed participants to build upon ideas and experiences shared within the group (Lyons and Coyle, 2007; Marques *et al*., 2020). Because of the nature of Zoom interviews, we kept the focus groups smaller than if we had held them in person. We also ensured that young people were given the opportunity to say if they would rather participate in single or mixed-gender focus groups or be in groups with siblings or friends. We also tried where possible to group participants of similar age together. Focus groups were led by SM, and were audio and video recorded via Zoom with the permission of young people and their parents. A Zoom transcription was generated and then checked for accuracy by members of the research team, which involved listening back to the focus groups while reading the transcription. This also contributed to the familiarization with the data.

The sections of the interview guide addressed in this article related to celebrity and SMI promotions of gambling and ways to respond. Examples of questions included: *‘What do you think about celebrities being in gambling ads?’; ‘Do you think young people are more likely to listen to an ad because a celebrity is in it, and why?’; ‘How do you think influencers might increase the appeal of gambling?’* Participants were also asked to consider some approaches that governments were taking in other countries to restrict the use of celebrities and SMIs in gambling advertisements. Photo elicitation methods were used to prompt discussion (Glaw *et al*., 2017). This enabled the researchers to discover different layers of meaning and elicit deeper insights and understandings from young people (Richard and Lahman, 2015; Glaw *et al*., 2017). Examples of the visual prompts included screenshots of promotions for online gambling that featured images of celebrities, tweets that featured popular influencers playing a poker tournament, and a video of a lottery promotion that featured a well-known Australian YouTuber. These were selected by members of the team based on information about marketing strategies provided by children in previous studies (Pitt *et al*., 2023; Thomas *et al*., 2023b), and to ensure that a range of different marketing strategies were presented to young people for comment. The participant information sheet highlighted to parents that gambling marketing would be shown during the interview. Some examples of the types of images their children would see were provided to parents so that they could make an informed decision about their child's participation. Data collection continued until there was sufficient quality, depth and richness of data in relation to young people’s attitudes towards the use of celebrities and SMIs in promotions, the influence of these types of promotions on young people’s gambling attitudes and consumption intentions, and the range of strategies that could be used to respond.

### Data interpretation

Data were interpreted using Braun and Clarke’s Reflexive Thematic Analysis (Braun and Clarke, 2020). Rather than using a software management tool, we conducted the analysis by hand and used Microsoft Excel to group data responses. Particular attention was given to critically engaging with the data to develop a deep understanding of young people’s responses. This included the team coming together to discuss interpretations and challenge any existing assumptions, thus enhancing the rigour of the research findings. Members of the research team familiarized themselves with the data by reading the focus group transcripts, with notes taken about the researchers’ initial thoughts and interpretations about the data. Active and thoughtful reflection occurred throughout to consider potential biases or preconceived ideas, with researchers constantly questioning the interpretation to explore deeper meanings and potential contradictions (Braun and Clarke, 2021). The research team met regularly to discuss and critically reflect on the interpretation of the data and ensured close attention was paid to the language that was used by the participants. Data were coded according to the research questions, focussing on both semantic (overt) and latent (nuanced) meanings, as well as identifying patterns and key concepts in the data. Themes were reviewed and refined by the research team to ensure they were distinct from each other before writing up the results. Quotes from participants were used to illustrate examples of the key themes and concepts.

## RESULTS

### Participant characteristics

A total of *n* = 64 young people aged 12–17 years participated in 22 focus groups which lasted between 60 and 90 min (Table 1). Most participants (*n* = 43) took part with at least one sibling in the same focus group, with the study involving young people from 22 families. About two-thirds of the sample were male (*n* = 44, 68.8%), over half were 12–13 years old (*n* = 33, 51.6%) and over half were from the state of Victoria (*n* = 38, 59.4%). Over two-thirds resided in areas of high socio-economic advantage (*n* = 43, 67.2%).

**Table 1:** Participant characteristics (*n* = 64)

	*n*	%
Age		
12–13 years	33	51.6
14–15 years	21	32.8
16–17 years	10	15.6
Gender		
Male	44	68.8
Female	19	29.7
Non-binary	1	1.6
State		
Victoria	38	59.4
New South Wales	26	40.6
SEIFA		
Low (1–3)	4	6.3
Middle (4–7)	17	26.6
High (8–10)	43	67.2

Four themes were constructed from the data. Supplementary File One provides a table of these themes and additional quotes to illustrate these themes.

### Celebrities and SMIs increase the appeal and recall of gambling promotions

Young people perceived that the use of celebrities or SMIs in gambling advertisements made people pay more attention to the advertisement than they normally would. Some young people discussed how seeing a familiar face would draw their attention and meant they would *‘actually listen to it’.* Young people commented that an advertisement featuring a recognizable celebrity could be more appealing for other young people and that they might find it more entertaining to watch than a normal advertisement. This was because it was attention-grabbing, made the advertisement more interesting, it was ‘*cool*’ to see a celebrity in an advertisement, and for one young person, it made them spend time trying to guess where they knew that person from. There was also a sense of familiarity with celebrities in gambling promotions, which some young people suggested would make the advertisement more memorable:

Because you recognise them, and when you recognise someone from an ad it makes it more interesting, and it makes you want to watch it more.—13-year-old girl, NSW

Participants commonly reported seeing ads that featured current or former athletes. For example, young people recalled gambling advertisements that featured sports stars such as ex-NBA athlete Shaquille O’Neil, and ex-Australian Football League players. Young people sometimes recalled that the promotions featuring these ex-athletes were aligned with particular sporting events or were played at different times during the match, with participants able to provide a detailed description of the content of these advertisements. Some young people particularly noted that some of these advertisements would prompt people to gamble because of the celebrity involved:

Before the footy and sometimes like during the breaks like half time, quarter time, there’s the Sportsbet ones with I think Nathan Brown does it, ah, up there with all the odds and like first goal kicker winners. Just all that, all those ads they pop out all the time. And yeah, just other random advertisements on TV.—13-year-old boy, Vic

When prompted, some participants recognized the SMIs in gambling promotions and social media content. In one focus group, the young people engaged in a discussion about recognizing the voice-over in a particular gambling promotion featuring an Australian YouTuber that *‘does funny videos’.* Participants recognized that his iconic voice made videos *‘sound really Australian’,* and thought that this would grab people’s attention:

It’s like soon as you hear the voice you just you switch over and look at it. So, if that was playing on the TV like then, that got my attention. So, I probably would have looked over. And because it’s Oz Lotto, it makes you pay attention, like go ‘oh, it’s Australian’.—17-year-old boy, NSW

### Celebrities and SMIs increase the trust, legitimacy and social acceptance of gambling

Young people stated that promotions featuring SMIs were particularly appealing to them because they were more relatable than celebrities. One participant stated that this was because SMIs seemed more like ‘*regular people*’. They explained that young people felt closer to SMIs because they were often part of their online communities, and consequently, they had more legitimacy than celebrities in the social media spaces that children and young people frequented:

It’s kind of like they’re more relevant to young people because they’re in spaces that are mostly inhabited by young people. So, Instagram, TikTok, etcetera. So, the influencers—it’s also like they’re more connected to me and my generation, what’s current and what’s relevant to me as opposed to the old people [who] don’t get you.—15-year-old boy, NSW

Young people also thought that the use of celebrities and SMIs in gambling advertisements increased the social acceptance of gambling. For example, some stated that celebrities or SMIs promoting gambling made it seem *‘okay to gamble’* and made it appear *‘acceptable to do’.* This included one 13-year-old boy who suggested that because SMIs were very culturally relevant it might *‘increase the appeal of gambling by showing how much more modern it’s becoming’.*

The use of celebrities and SMIs also added trust and credibility to the gambling company or gambling product that they were advertising. Some young people discussed how athletes in particular added *‘an extra level of trust’* when promoting sports betting. This was because they perceived that athletes would be knowledgeable about the sport that they were promoting. For example, participants discussed that former NBA player Shaquille O’Neal would have *‘expertise’* in sport and *‘know what he’s talking about when it comes to betting on basketball or sports in general’.* However, some were concerned about the level of trust that celebrity and SMI promotions could create in gambling companies and brands, and the particular impact of this on children and young people. This was thought to lead young people to believe that gambling was a safe and acceptable activity because it created the perception that the celebrities were also using the product:

I reckon like younger audience like late teens, early 20s would see him like this and probably go if Shaq [Shaquille O’Neal] is in an ad for Pointsbet, he must use them. Meaning they’re probably a good and trustworthy company, and they must have benefits to them. So, I reckon it would pull in more of the younger audience or people that like know him.—12-year-old boy, Vic

Young people also stated that children looked up to, idolized and aspired to be like celebrities. In one focus group there was a discussion about a basketballer who was featured in gambling advertisements, with one participant commenting that they were currently wearing the athlete’s jersey:

13-year-old girl, NSW: Well, um, the basketball player, so many little kids who are my brother’s age, they all watch basketball and look up to him because he’s a basketball player, a really good one.

12-year-old boy, NSW: I’m wearing his shirt right now.

Participants commented that the perception that people whom they idolized were gambling could also encourage young people to try gambling—*‘when they see them getting involved in betting, they will probably do the same as well’*. One young person specifically described the impact of SMIs on those who were young but were at a legal age to gamble:

A lot of influencers’ audience are younger like under 20s, like up to 18, 19-ish. So, if they show off gambling... for the people in the, within the legal age, they’ll probably go, ‘Well, if my idol, my favourite YouTuber, Instagrammer, TikToker is gambling maybe I should give it a try’.—12-year-old boy, Vic

Participants also perceived that having a celebrity or SMI in a gambling advertisement would make the advertising more convincing for young people and would encourage them to gamble—*‘why can’t I be like them?’* Others suggested that celebrities would make it more tempting to try gambling than if there were no celebrities:

It also is like tempting young people as well because like young people when they see like the ad without the YouTuber, they wouldn’t think it’s as like cool and um like it’s not as tempting to do. But as soon as they see a famous YouTuber or TikToker or Instagram like they think that suddenly it’s cool and they’re doing it, so they want to be just like what they’re doing.—13-year-old girl, NSW

Finally, some young people suggested that because celebrities and SMIs lived aspirational lifestyles, their appearance in gambling advertising may give viewers the impression that *‘gambling might help them achieve that’* same lifestyle. For example, some participants noted that celebrity lifestyles of wealth and glamour were often portrayed in gambling advertisements and gave the impression that the celebrity was wealthy because of gambling. Others suggested that gambling would help you attain the same lifestyle as these celebrities:

I think you kind of get shown a bit of their lifestyle in it and think that if you can um win like they are then, um, then you can like have that lifestyle that they’re living in the ads.—15-year-old boy, Vic

### Celebrities and SMIs lower perceptions of the risks associated with gambling

Some young people commented that celebrity and SMI promotions could create a perception that gambling had little risk attached to it, or that gambling would always lead to a win. Many specifically commented that there was mostly positive commentary about gambling, particularly from SMIs, with very limited emphasis on losses. Participants explained that these types of promotions, particularly involving SMIs engaging in gambling on some platforms, created an unrealistic view of the outcomes of gambling:

They’re kind of just never like—pretty much all ads don’t really focus on the negatives. They’re just saying, ‘Oh, I won this much’ and they wouldn’t really say ‘But I almost lost it’ or something like that.—12-year-old girl, Vic

Young people were sceptical about whether the celebrity or SMI had any genuine relationship with or interest in gambling or rather were only promoting this because they had been paid to do so. This was considered very inauthentic by young people where the influencer was seen as ‘*not caring about the subscribers*’ or the well-being of others. Contributing to this was confusion regarding whether posts were paid or sponsored. For example, many young people were concerned about the lack of transparency associated with SMIs who showed themselves gambling online. Participants were sceptical as to whether they could trust the authenticity of gambling content online if SMIs were being paid to promote it, saying that this would result in SMIs talking only about the positive side of gambling. Other young people spoke about how paid endorsements would be ‘*all set up for them*’ to win, not showcasing the true reality of gambling. Participants suggested that when celebrities or SMIs gambled it was manipulated to ensure they always won for the purpose of influencing:

It’s definitely kind of like sharing a fake story. Like they’re acting like it’s they’re just gambling but they’re getting paid. It’s all set up for them. It’s not at all like what real gambling is like so it’s kind of like tricking you into wanting to do it.—16-year-old girl, Vic

Some young people criticized celebrities and SMIs for promoting gambling, stating that they already earned a lot of money and that these types of sponsorships were unnecessary and ‘*disheartening’*:

I think personally that when celebrities like do participate in ads for gambling, they’re not thinking about the wellbeing of others. They’re just thinking about the money that they’re going to earn through doing the ad. They’re not thinking about the young people that are watching it. So, I don’t really think that they should be participating in these ads.—13-year-old girl, NSW

### Reducing the impact of celebrity and SMI gambling promotions on young people

Young people offered a range of suggestions regarding how to reduce the impact of celebrity and SMI gambling promotions on young people. Some suggested that celebrities and SMIs should take personal responsibility for their role in these promotions and turn down paid gambling deals. This was especially the case if the celebrities and SMIs *‘know they have a young, impressionable audience’.* Participants also recommended that celebrities and SMIs should get involved in anti-gambling campaigns or use their platforms to provide risk messaging about gambling. For example, one 17-year-old boy stated that some celebrities could push against other celebrities that endorse gambling and *‘fight fire with fire’.* However, many participants suggested that the complete banning of celebrities and SMIs in gambling advertisements was needed to protect young people and *‘make it less influential to the young generation’.* They argued that banning celebrity and SMI involvement in gambling promotions would prevent young people from paying attention to gambling promotions, and that young people would subsequently lose interest in the content of the advertising. Some young people believed that removing celebrities and SMIs from these promotions would also reduce the number of young people who knew about gambling:

I think if you’re encouraged more at a younger age, you get a better understanding when you’re younger so by the time you’re like 20 or something, you’ll have like a really good understanding which also can be dangerous. So, I think if they like reduce the amount of young people who know about gambling, it’s going to help reduce the negative impacts in this.—16-year-old girl, NSW

While some young people believed that a complete ban of celebrities and SMIs in gambling promotions would be appropriate they also thought that it would be hard to enforce. Participants suggested that governments might have difficulties in classifying celebrities and SMIs, and that gambling companies would find ‘*loopholes*’ or ways to bypass any regulations that were made. Some young people argued that more comprehensive regulations on all forms of gambling marketing were needed to protect children and those who might be vulnerable to gambling harm. Some advocated for complete bans on all gambling marketing to prevent people from gambling in the first place, and to protect people from harm:

People may be falling into a bad place because they got addicted to gambling and governments want to help prevent this because governments are supposed to be good people, right? Even though some of them aren’t, they still want to make people happy because that’s the way they stay in power.—13-year-old boy, NSW

However, some young people were sceptical that governments would implement comprehensive restrictions on gambling, stating that Australian governments had a long history of ineffective gambling advertising regulation, and were heavily influenced by the gambling industry:

Although I don’t think that it would ever happen in Australia, simply because um the government is heavily influenced by the gambling industry as it is with any other industry that will bring them in money.—15-year-old boy, NSW

## DISCUSSION

This research aimed to provide an in-depth understanding of young people’s views about the impact of using celebrities and SMIs for the promotion of gambling. While marketing theorists have discussed the influence of celebrities and SMIs in transferring symbolic meanings to products and brands (McCracken, 1989; Engel *et al*., 2024), this study aimed to understand how this may be occurring for gambling from the perspectives of young people. This study extends current gambling research which has quantified the extent and nature of different types of marketing appeal strategies (including on social media) (Pitt *et al*., 2023), and the impact of television and sports-based gambling marketing on young people (Djohari *et al*., 2019). The study sought to document how young people describe the influence of specific types of novel marketing strategies. The findings show that, from the perspective of young people, the use of celebrities and SMIs is an effective strategy used by the gambling industry to bring attention to gambling advertisements, increase trust in gambling and gambling brands and reduce perceptions of the risks associated with gambling products and engaging in gambling. The study also demonstrates the importance of ensuring that research investigating the impact of gambling marketing strategies on young people takes into account how young people are exposed to marketing via a range of media channels outside of traditional television advertising or sporting environments (Thomas *et al*., 2023b).

Hudders and Lou, (2023) note that SMIs are able to seamlessly integrate marketing into their content and have an ability to engage their audience in a way that interacts with their content. They go on to argue that young people are particularly vulnerable to SMI marketing because they lack the ability to critically reflect on the content that they are seeing. The findings from this research suggest that SMI marketing may pose additional risks as compared to other forms of gambling marketing because of the connection that influencers have with young people. This includes that young people see themselves as part of the SMIs’ community and feel that they have a connection with the SMI. They may also be influenced by the selective portrayal of positive outcomes in SMI content related to gambling, including through the portrayal of the positive impacts of gambling such as winning money without addressing the potential risks or losses. This observation indicates that as SMIs tend to present an idealized and curated version of their lives, this may not accurately reflect the realities and potential negative consequences of gambling. These types of marketing strategies could be classified as agnogenic, designed to create ignorance about the realities of the risks and harms associated with gambling products.

This can also be extended to young people’s confusion about whether gambling content involving SMIs was a ‘promotion’ or not. For example, broader marketing research shows that young people are often unaware that sponsored posts by SMIs are advertising (De Jans *et al*., 2020). However, Borchers, (2022) notes that rather than being unaware of marketing strategies, there may instead be a tension for young people who on the one hand may understand that what they are seeing is marketing, but on the other hand, may also identify and relate to the SMI. The use of SMIs for the promotion of gambling (and other harmful products such as vaping and alcohol) therefore may represent a heightened risk to young people, particularly if they are unable (or unwilling) to recognize the persuasive intent of SMI gambling promotions as compared with traditional advertising. This study also suggests that we may be seeing similar patterns of engagement with SMI marketing across a range of harmful industries. For example, alcohol researchers have argued that while young people may feel a personal connection to SMIs, the lack of offline relationship with the SMI may make it difficult for young people to critically evaluate SMI content for realism and authenticity (Corcoran *et al*., 2023). Developing integrated approaches to research and policy which consider and examine how these strategies may be used and applied across a range of industries will be important in progressing comprehensive policy frameworks which focus on harmful marketing tactics as well as responses to specific industries or products.

This study also provides evidence that young people are able to provide new perspectives on issues that may not have been considered by researchers or policy makers regarding novel gambling promotions, and provide suggestions for how to respond to such issues. Previous public health initiatives have clearly demonstrated the important perspectives young people can bring to policy discussions about harmful industry tactics (Pitt *et al*., 2022; Soraghan *et al*., 2023), and that they should be treated as genuine political citizens in responses to these issues (Arnot *et al*., 2023a). Young people in this study were supportive of increased restrictions and regulations on gambling marketing, increased transparency about the risks associated with gambling, and more inclusion of SMIs in gambling harm prevention campaigns focused on industry tactics and products. However, as has been seen in other studies which have canvassed young people’s views about government interventions on harmful industries (Arnot *et al.*, 2023b), young people are sceptical about the likelihood of governments acting in their best interests. This concern appears well-founded given evidence showing how harmful industries have used social media platforms to circumvent regulations on their marketing and to reach new youth audiences (Kong *et al*., 2022; Campaign for Tobacco-Free Kids, 2023) with limited adequate response from governments to prevent this. While marketing through these platforms may not have the same visibility for policy-makers and regulators as traditional advertising, the findings from this study suggest that there should be also be increased surveillance and monitoring of how harmful industries are using these platforms to reach their next generation of consumers, with the appropriate regulatory responses implemented to protect children.

### Limitations

There were a number of limitations in the study. First, there was a gender imbalance in the sample with the majority of participants being male. This may be due to the fact that the study relied on parental referrals and may reflect parents being more concerned about the potential influence of gambling advertising on boys than on girls. Parents may also perceive that boys are more exposed to gambling content and may have more to say on this topic. Second, there was an overrepresentation of individuals from high socio-economic status (SES) backgrounds. Participants with higher SES may have different experiences and attitudes towards gambling advertising compared with those from lower socio-economic backgrounds. Researchers should ensure that the experiences of young people from diverse backgrounds are included in such studies. The sampling and recruitment strategy for this study, for example, through social media networks, sporting organizations and excluding those fluent in English may also have impacted on the skew towards individuals from high SES backgrounds in the study. Finally, young people were recruited from Victoria and NSW. These are the states with the largest gambling losses in Australia, and have their own distinct gambling environments and regulations. Responses from young people in other states and territories may be different to those in this study.

## CONCLUSION

Regulatory efforts to protect children and young people from gambling marketing should be part of a comprehensive public health approach to prevent gambling harm. This should also include legislation, strong public education campaigns and policies that restrict gambling accessibility and availability. Given the urgency of the issue, it is imperative that governments take immediate and effective action to protect the next generation from gambling harm. By implementing evidence-based public health measures, policymakers can not only protect young people but also benefit the broader community. Action to protect their next generation from gambling harm should include a special focus on monitoring and regulating all forms of gambling marketing, including novel approaches designed to influence young people.

## Supplementary Material

daae012_suppl_Supplementary_Material
