# Supplementary material for: Young people’s views about the use of celebrities and social media influencers in gambling marketing
Source: Health Promot Int. 2024 Feb 11;39(1):daae012. doi: 10.1093/heapro/daae012 (PMC10859070; doi:10.1093/heapro/daae012)
Supplement: daae012_suppl_Supplementary_Material [file daae012_suppl_supplementary_material.docx]

**Supplementary File One: Summary of key themes and additional quotes**

| **Themes** | **Sub themes** | **Quotes** |
| --- | --- | --- |
| *Celebrities and SMIs increase the appeal and recall of gambling promotions* | Paying more attention | I think having [a] celebrity there probably, um, they're more aware of the ad because they have a recognizable face. 17-year-old, Female, Vic  Well, to some aspect, it depends on how well known the celebrity is. For example, I see the Pointsbet one, the Shaquille O'Neal, and I go, “well, Shaq sponsors gambling” or something. I don't really care that much. But if you were heavily into basketball, that would probably be quite a big deal. You’d care a lot more. 16-year-old, Male, Vic |
|  | Recall of sports stars and ex athletes | Well, there are those ads, there are those ads of like celebrities playing a game, using the betting apps, and it’s like oh, you can do – like Shaq and he's like “Oh, I can get a back massage while betting.” And like it's a kind of showing celebrities, like, the celebrities do it, you can do it, too. Like it's so easy and those ads to people being like “Oh, look it's like James Bond level technology” and they're like, “Oh look, we've developed a new thing to the app.” 16-year-old, Female, Vic  I know a few that have celebrities in it. Like I’ve seen the ones that have like Shaquille O’Neal and then everybody who thinks he's cool then thinks that they should go gamble and then they lose a lot of money. 12-year-old, Male, Vic |
|  | Recognised celebrities and SMI | Facilitator: Participant X what do you think would be the most appealing to young people? 17 year-old, Female, Vic: Um, probably like the KSI and Logan Paul. Like a familiar face a lot of kids know about them. Yeah.  Facilitator: What gambling ads you have seen? 17-year-old, Male, NSW: You see the like Ladbrokes ones where you got Mark Wahlberg coz he's like a familiar face, so people take to it. |
| *Celebrities and SMIs increase the trust, legitimacy and social acceptance of gambling* | Relatable and legitimate | I find a lot of influencers seem like relatable or just fun people to be around, just if you were to know them in person. So, um so having them promote something will make it seem like, well, they’ll think it’s a good thing. As I mentioned earlier, quite a lot of people look up to these content creators and influencers. So, they would go through whatever it is that they're telling them to do. 13-year-old, Male, NSW |
|  | Social acceptance | By the very nature, influencers are seen as very culturally relevant so if they’re doing this, it might be more “hey, this is something that’s acceptable to do and we can do it” and like that. And it raises – like TV ads with most people migrating to the small screen of the phones and computers, it’s a much more effective way of doing that. And with influencers and gambling, it’s kind of happening. 15-year-old, Male, NSW  I know that some people use like products that the celebrities use, so, I guess if gambling was like normal to [a] celebrity, kids would probably try to follow in the footsteps. 17-year-old, Female, Vic |
|  | Trust and credibility | I think it has a big impact on the people who look up to them. And they go, “okay well he's part of – or she’s part of it, I’ll do that as well.” 13-year-old, Male, NSW |
|  | Idolised | It’s not good coz mostly young people watch those YouTubers and then they look at them and it shows them that it’s okay, everyone does it. And even like the people you idolize do it so, and you want to be like them, so you do it. 13-year-old, Female, Vic  yeah because people follow [celebrities], people look up to them and idolise them and so they’ll practically do what they say to do. 14-year-old, Male, Vic  Especially if they’re someone's idol, like you really look up to them and what they do. So, if they're promoting [gambling] then that's something that you would look into and, like I guess yeah, whatever they do you kind of look up to, so yeah. 16-year-old, Female, NSW |
|  | Aspirational | Their lifestyles like seem to be very like prestigious and like really cool. And they make it seem like they've got a lot of things going on and like this is how you should live your life. And I think young people act to follow people like that and like to kind of be influenced by how they live and what they do. 16-year-old, Female, NSW  People see someone who's obviously rich and famous, and they aspire to be like them and they're like, “oh, you know if they use this gambling thing then maybe that's how you can be like them”, you know. 16-year-old, Male, Vic |
| *Celebrities and SMIs lower perceptions of the risks associated with gambling.* | Reduced level of risk | I think that when influencers do these types of videos there's always something to be won but it's just smaller in value than the biggest prize and so people get the wrong idea. And the misconception that you will win something, no matter how much you put in you'll always get something out of it which isn't right when it comes to gambling. 15-year-old, Female, Vic  Usually people who have a, like bigger platform like YouTube and like Instagram, TikTok, stuff like that, I think they have a more effective way of gathering people to have different opinions or the same opinions as that person. So then this person might make you think that like, gambling is only fun and gambling is only winning, but yeah it's not really like that in reality. 13-year-old, Male, Vic |
|  | Scepticism around the messages and lack of transparency | 12-year-old, Male, NSW: These companies aim for young people coz they know their minds doesn't know what it's about. Yeah, they just think everything's like, “Oh, they will never lie to me. Oh, let's do this.” They’re young so they don’t fully know – well, I’m young but I know what can happen. Facilitator: Yeah so, maybe other people are quite susceptible because they don't know what's being paid or things like that. 12-year-old, Male, NSW: Yeah. That’s what I mean. Exactly that.   They're kind of just never like – pretty much all ads don’t really focus on the negatives. They’re just saying, “Oh, I won this much” and they wouldn’t really say “But I almost lost it” or something like that. 12-year-old, Female, Vic |
| *Reducing the impact of celebrity and SMI gambling promotions on young people* | Using celebrities and SMIs in messages that counter gambling advertisements | Uh, I actually agree with Participant X. I reckon that would be a really good idea with, if you got like, there's a lot of celebrities who are going towards gambling. But they're not- there's not really that many that are going against saying “you should stop gambling”. And I reckon if they got famous people to do that it would like, influence them say, it's like if they did say that it’s good to gamble, “oh this’d be good”, but if they said it’s not good to gamble, they’d be like, they’d say, “oh this person says it not good, we shouldn’t do it”. 13-year-old, Male, Vic  Maybe getting influencers to advertise for anti-gambling. You know, just sticking up for the addiction of gambling, I think would be a practical way of reducing the gambling addiction. 15-year-old, Male, Vic  I suppose you could probably try to get more influencers, so advertise anti-gambling instead of promoting it. 15-year-old, Male, NSW |
|  | Banning celebrities and SMI from gambling advertising | In general, yes, it's a good idea. I mean not for the companies but like influencers are generally appealing to the younger community, so it will help stop. 15-year-old, Male, NSW  The more influencers endorse these brands and gambling, the more people are likely to go and gamble, and so if no celebrities are seen gambling or endorsing gambling, then less people are going to end up gambling and losing all their money or winning it. 14-year-old, Male, Vic  I think [banning celebrities and SMI from gambling advertising] will definitely make younger people not want to, it would make less people see gambling and also see gambling as more the bad idea, more than a good idea because celebrities mostly win in the ads or are very happy. 12-year-old, Male, NSW |
|  | Difficult to regulate and hard to enforce | I reckon they’d have to do something a bit more major before people would really kind of stop thinking gambling was such a good idea. 13-year-old, Male, NSW  I kind of remember them doing something in Australia I don’t know if it lasted but wasn't there a rule years ago, the gambling ads couldn't be shown until like 8.30 when all the kids are asleep. They couldn't play gambling ads during like big sporting events because kids would be watching but I don’t think that one held up because I see gambling ads during sporting matches all the time. 15-year-old, Male, Vic  I don’t think it’s worth the effort. If they’re going to try and do things about gambling, they should put much stricter regulations on it or just outright make it illegal instead of doing this kind of thing. 13-year-old, Male, NSW |
